# Irrigation and Nitrogen Fertilization Alter Soil Bacterial Communities, Soil Enzyme Activities, and Nutrient Availability in Maize Crop

**DOI:** 10.3389/fmicb.2022.833758

**Published:** 2022-02-03

**Authors:** Ihsan Muhammad, Li Yang, Shakeel Ahmad, Muhammad Zeeshan, Saqib Farooq, Izhar Ali, Ahmad Khan, Xun Bo Zhou

**Affiliations:** ^1^Guangxi Colleges and Universities Key Laboratory of Crop Cultivation and Tillage, College of Agriculture, Guangxi University, Nanning, China; ^2^Department of Agronomy, University of Agriculture, Peshawar, Pakistan

**Keywords:** bacterial community, bacterial diversity, soil enzymes, physio-chemical properties, irrigation

## Abstract

Irrigation and nitrogen (N) fertilization rates are widely used to increase crop growth and yield and promote the sustainable production of the maize crop. However, our understanding of irrigation and N fertilization in the soil microenvironment is still evolving, and further research on soil bacterial communities under maize crop with irrigation and N management in subtropical regions of China is needed. Therefore, we evaluated the responses of two irrigation levels (low and high irrigation water with 60 and 80% field capacity, respectively) and five N fertilization rates [i.e., control (N0), N200 (200 kg N ha^−1^), N250 (250 kg N ha^−1^), N300 (300 kg N ha^−1^), and N350 (350 kg N ha^−1^)] on soil bacterial communities, richness, and diversity. We found that both irrigation and N fertilization significantly affected bacterial richness, diversity index, and number of sequences. Low irrigation with N300 treatment has significantly higher soil enzymes activities, soil nutrient content, and bacterial alpha and beta diversity than high irrigation. In addition, the Proteobacteria, Actinobacteriota, Chloroflexi, and Firmicutes were the dominant bacterial phyla under both irrigation regimes. The acidic phosphates, acidic invertase, β-glucosidase, catalase, cellulase, and urease were positively correlated with the Shannon index under both low and high irrigation. Therefore, low irrigation improves soil nutrient utilization by boosting soil enzyme activity, directly affecting soil bacterial communities. It was concluded that greater soil nutrients, enzyme activities with higher bacterial diversity are the main indicators of soil reactivity to low irrigation water and N300 for maintaining soil fertility and soil microbial community balance.

## Introduction

The global demand for greater yield while maintaining environmental quality is a major challenge, particularly in the maize cropping system (Cassman et al., 2002). Irrigation water and nitrogen (N) fertilizer are the two most important leading inputs affecting agricultural cropping systems (Gheysari et al., 2009). Nitrogen fertilization is one of the most important factors influencing crop growth and development. To produce high yields, extensive water and fertilizer inputs are typically used (Wang et al., 2019). Excess fertilization and poorly managed irrigation regimes, on the other hand, are common practices that cause soil and water environmental concerns (Mack et al., 2005; Gheysari et al., 2009; Jia et al., 2014; Huang et al., 2017), resulting in a 20% N contribution to the environment as a result of farming practices (Jones and Downing, 2009). Regular N fertilization is required to ensure optimal yields, especially in high-yield crops like maize (*Zea mays* L.). On the other hand, the desire for food compelled the development of new farming practices, which disrupted the N cycle within the agroecosystem (Pelzer et al., 2012). There is a concern about optimizing the fertilization strategy, because only a minute fraction of applied mineral fertilizer is absorbed by plants, and almost 30%–50% of these nutrients can be lost in different ways (Jensen et al., 2012).

Many factors influence the bacterial community in soil, such as soil physio-chemical properties and soil enzyme activities, and other anthropogenic inputs, including fertilization, organic carbon management, irrigation regime, and irrigation frequency (Frenk et al., 2015, 2018). The impacts of these inputs on the bacterial community are deemed stochastic factors, which are also referred to as “habitat filters” or “niche based” in the literature (Dini-Andreote et al., 2015). Zeng et al. (2016) found that the abundance of soil microbes and the activity of enzymes in soil can be significantly affected by nitrogen fertilizer and irrigation regimes (Ming et al., 2018). Soil enzymes and microbial populations are vital to the long-term viability of soil ecological activities, as they are directly involved in energy flow, nutrient cycling, and organic matter decomposition (Hou et al., 2020; Khan et al., 2021). The microbial community is a key indicator for assessing soil strength and quality. Soil microbial populations are extremely susceptible to soil disturbances such as tillage and irrigation water (Frenk et al., 2014). Soil microbes regulate C and N cycles and serve as key nutrient repositories, therefore maintaining their complexity and diversity is vital for soil fertility (Trivedi et al., 2017). Irrigation and fertilizer are the two most important agricultural management strategies in crop production because they boost crop growth, development, and yield while also influencing the soil microbial community (Feng et al., 2015). As a result, it is critical to investigate the effects of N fertilizer in conjunction with irrigation on the diversity, population, and community structure of soil microorganisms.

Nitrogen fertilization affects bacterial community compositions because it provides a direct supply of nutrients to soils while also changing soil pH and stimulating the decomposition process (Beare et al., 2002). Muhammad et al. (2018), reported that nitrogen is the most abundant macro element in soil, and inadequate nutrient cycling in ecosystems is a prevalent problem that has been extensively investigated. Nitrogen fertilizer is a widespread agricultural strategy used to boost crop yield and soil biogeochemical cycles; however, excessive application can lead to soil acidification, ecological issues, pollution, and loss of ecosystem biodiversity (Yue et al., 2019; Zhao et al., 2019). With the widespread use of chemical fertilizers, farmland ecosystems are witnessing historic increases in nutrient inputs. However, unbalanced fertilization might have a considerable impact on farmland ecological ratios, as well as plant and soil elemental ratios, and ecosystem diversity (Liu et al., 2020). Therefore, for improved maize yield, soil fertility, enzyme activities, and soil bacterial diversity, one needs to focus on N fertilization and irrigation management.

In the soil ecosystem, microbial communities play a critical role in nutrient cycling, decomposition, the maintenance of soil health, and boosting soil enzyme activities (Wang et al., 2021b). According to a study by Zeng et al. (2016), dehydrogenase activity and the population of Actinobacteria did not improve when high nitrogen doses were applied to maize fields and had an adverse impact on soil microbes (Muhammad et al., 2021a). Sawicka et al. (2020) suggest that root exudates are a great source of nutrients for bacteria, particularly those residing in the rhizosphere., As a result, mineral nitrogen fertilization increases the population of bacteria in the soil because of the increased amount of nitrogen in the soil and changes in its physio-chemical properties (Burns et al., 2013; Muhammad et al., 2021b). Fertilization provides the essential nutrients for plant growth and development and improves soil quality by increasing nutrient absorption rates (Hou et al., 2020). Long-term fertilizing altered soil enzymatic activity and physio-characteristics, such as soil pH, total carbon, nitrogen, and phosphorus, potentially resulting in major variations in soil microorganisms (Li et al., 2021). However, Li et al. (2021) revealed that fertilization had no influence on the composition and structure of the bacterial community, as well as on bacterial diversity, because fertilization simply raised the content of soil organic carbon (SOC) and had no effect on the soil pH level. Consecutive monoculture is a common practice in the maize crop. Limited studies have been conducted on the selective enrichment of microbial communities through different nitrogen fertilizer rates and irrigation regimes. Though microbial community structures differ among plant species, informative studies concerning the distinctness of microbial communities in different irrigation regimes are lacking. As a result, our research focused on: (1) high throughput sequencing to investigate the soil bacterial communities in maize crop, (2) to study the soil bacterial response to different N fertilizer rates and irrigation, and (3) their effect on soil enzymes and physio-chemical properties and (4), to elucidate the mechanism of action of N fertilizer and irrigation on microorganisms.

## Materials and Methods

### Experimental Site

A pot experiment was conducted at Guangxi University in the subtropical region of China (22°50, 108°17) in a greenhouse with a controlled atmosphere. The Köppen-Geiger climate classification for the area is Cfa, indicating a warm and temperate climate. The average annual temperature and rainfall are 21.8°C and 1,662 mm, respectively, with a 227 mm variation between the driest and wettest months. Temperatures fluctuate by 14.7°C throughout the year, with July being the warmest and January being the coldest, with average temperatures of 27.7 and 13.0°C, respectively. The soil texture was clay loam according to Chinese Soil Taxonomy with a pH of 6.5, field capacity of 44%, soil bulk density of 1.40 g cm^−3^, soil organic matter of 20 g kg^−1^, and available nitrogen, phosphorus, and potassium of 127.0, 40.0, and 126 mg kg^−1^, respectively. A pot was filled with soil gathered from the greenhouse, which had been dormant for the previous 6 years.

### Experimental Design and Management

The experiment was carried out in a completely randomized design with three replications in a controlled-environment greenhouse in Guangxi, China. The experimental treatments were two irrigation levels, such as low irrigation water with 60% field capacity and high irrigation water with 80% of field capacity, and five nitrogen rates, such as control (N0), N200 (200 kg N ha^−1^), N250 (250 kg N ha^−1^), N300 (300 kg N ha^−1^), and N350 (350 kg N ha^−1^). The five uniformly sized hybrid maize seeds (Zhengda 619) were planted on 28 September 2020 and harvested on 8 April, which is the most commonly grown variety in the subtropical areas of China. The seeds were obtained from CP Seed Industry, Yunnan Zhengda Seed Co. Ltd., China. The selected seeds permission was granted by the respective authority. The base fertilizers (P and K) and 1/2 of N were thoroughly mixed with the soil before sowing, and the remaining 1/2 of N was applied as a top dressing at the nine-leaf stage. The phosphorus (P) and potash (K) fertilizers were used in accordance with local fertilization standards, at 100 kg P ha^−1^ and 100 kg K ha^−1^ to ensure that all experimental treatments had equal P and K concentrations. The fertilizer types used in our experiment were urea (46% N), phosphorus pentoxide (P_2_O_5_; 18% P), and potassium oxide (K_2_O; 60% K). Maize crops are trimmed to four plants per pot at the three-leaf stage to facilitate better adaptability to the pot environment. Throughout the growth stage, plants were watered with tap water to maintain soil moisture at 60 and 80% of the field water holding capacity, respectively.

The homogenized soil sample was taken from the plant rhizosphere of each pot at the end of the experiment, and roots and other debris were removed. Half of the soil samples were air–dried, sieved, and used for the determination of soil total nitrogen (STN) using Kjeldahl procedure and SOC by K_2_Cr_2_O_2_ extraction method, while the other half of the soil samples were kept in a refrigerator at −80°C until the determination of soil ammonium (SNH_4_^+^-N) and nitrate (SNO_3_^−^-N) and molecular analysis. Additionally, the soil samples were air–dried at room temperature and sieved through 0.069 mm mesh for enzyme activity analysis.

### Measurement of Soil Enzyme Activities

The activities of soil acid phosphatase (S_ACP; BC0145), acid invertase (S_AI; BC3075), β-glucosides (S_β-GC; BC0165), catalase (S_CAT; BC0105), cellulose (S_CL; BC0155), and urease (S_UR; BC0125) were determined using the soil enzyme kit from Solarbio Science & Technology Co., Ltd. (Beijing, China). The methods of determination are described in detail in the manual.

### Soil Organic C, N, and pH Analysis

Potassium dichromate volumetric and external heating techniques were used to quantify SOC (Chen et al., 2021), while soil TN was measured by the semi-micro kelvin method (Wang et al., 2021a). Using an electronic pH meter, the soil pH was determined potentiometrically (1:5 suspensions) using the method of Rhoads (1996).

### DNA Preparation, PCR Amplification, and High-Throughput Sequencing

Total genomic DNA was extracted from each soil sample according to the manufacturer guidelines using the E.Z.N.A® DNA Kit for Soil (Omega Bio-tek Inc., Norcross, United States). The final extracted DNA concentrations were measured using the NanoDrop 2000 (Thermo Fisher Scientific, Wilmington, United States). The DNA samples served as a template for the amplification of the bacterial rRNA gene. The 338F (forward primer, 5-ACTCCTACGGGAGGCAGCAG-3) and 806R (reverse primer, 5-GGACTACHVGGGTWTCTAAT-3) primer sets were used for amplification of the bacterial V3–V4 region of 16S rRNA (Chen et al., 2018). The PCR was carried out in a total volume of 50 μl, which included 25 μl of 2 × Premix Taq (Takara Biotechnology, Dalian Co., Ltd., China), 1 μl of each primer (10 mM), and 3 μl of DNA (20 ng μl^−1^) template, and added sterilized deionized water to make the volume up to 50 μl. Following a 5-min denaturation at 94°C, 30 cycles of denaturation at 94°C for 30 s, annealing at 52°C for 30 s, extending at 72°C for 30 s, and finally elongating at 72°C for 10 min were performed.

### Processing of Illumina Sequencing Data

Majorbio Bio-Pharm Technology Co., Ltd. (Shanghai, China; Zeng et al., 2020) extracted the bacterial PCR products, pooled them in equimolar quantities, and sequenced them (2 × 300) on an Illumina MiSeq platform (Illumina, San Diego, CA, United States; Zeng et al., 2020). The processed sequences were clustered into operational taxonomic units (OTUs) with at least 97% similarity using UPARSE version 7.1 (Gdanetz et al., 2017). To test the taxonomy of bacterial sequences against the SILVA database version 128/16S-bactera database, the RDP Classifier method was employed with a confidence level of 70% (Zheng et al., 2020).

### Alpha and Beta Diversity Analysis

The bacterial diversity in each sample was assessed using an OTU-based analytical approach. The OTU richness (Chao1) and diversity (Shannon indices) were calculated using QIIME software (v1.8.0) with a sequencing depth of 3% to assess the diversity index and species richness (alpha diversity) of both irrigation and N fertilizer for each sample. The OTUs’ 97% similarity was used to create the rarefaction curves.

For the comparison index estimation of the community structure, beta diversity analysis was employed across all samples. Beta diversity was computed at the OTU level of genotypes using weighted UniFrac distances and was visualized using principal coordinates analysis (PCoA). The weighted UniFrac distance matrices were grouped and analyzed using the QIIME program (v1.8.0). They revealed phylogenetic relationships between diverse communities and their abundance in the relevant samples.

### Statistical Analyses

Statistical data from soil environmental parameters and soil enzyme activities were analyzed and compared using SPSS22.0 analysis of variance and multiple comparisons (SPSS, Chicago, IL, United States). The Chao1 richness index and Shannon diversity index were used to calculate alpha diversity. The correlation between soil enzymes activities and alpha diversity was done with the R (version 3.6.3) package “corrplot” (Wei et al., 2017). The Bray-Curtis distance matrix was used to estimate beta diversity using PCoA. We also performed the Mantel test to investigate the link between beta diversity and soil enzymes using “vegan” packages in R v3.6.3 (Oksanen et al., 2013). The impact of soil compartments on OTU abundance was studied with edge R (Robinson et al., 2010) on TMM-normalized data (Robinson and Oshlack, 2010) and illustrated with ternary plots. The Spearman rank correlations between bacterial phyla were calculated using TMM-normalized data.

## Results

### Soil Physio-Chemical Properties and Enzyme Activities

The irrigation and nitrogen fertilization significantly affected soil enzyme activities (Table 1). The results showed that the low irrigation regime had significantly higher acid phosphatase, acid invertase, β-glucosidase, catalase, cellulose, and urease activities. In addition, STN, SOC, SHN_4_^+^-N, and SNO_3_^−^-N were also significantly increased under low irrigation with N300 treatment, but irrigation had no significant effect on soil pH level. The acid phosphatase and β-glucosidase were significantly higher at N350 compared to other N treatment, however not statistically different from N300 treatment. Furthermore, the acid invertase, catalase, cellulase, and urease activities were significantly higher at N300 treatment, and the lowest activities were observed at N0 treatment. The correlation analysis revealed that the SNO_3_^−^-N, SNH_4_^+^-N, SOC, and STN were significantly and positively correlated with each other, as well as with soil enzyme activities, while significantly negative correlated to soil pH (Table 2). Similarly, the soil acid phosphatase, acid invertase, β-glucosidase, catalase, cellulase, and urease activities were also highly positive and significantly correlated with each other and negative correlated to soil pH. The STN ranged from 14.83 to 19.18 g kg^−1^, whereas the highest STN was observed for N350 and the lowest was observed for N0 (Table 1). Furthermore, these results suggested that irrigation and N fertilization both had a strong and significant effect on soil physio-chemical properties.

**Table 1:** Effects of irrigation water and N fertilizer rates on soil enzymes and physio-chemical properties in maize crop.

Irrigation	Nitrogen	S_ACP	S_AI	S_β_GC	S_CAT	S_CL	S_UR	STN (g kg^−1^)	SOC (g kg^−1^)	SNH_4_ (mg kg^−1^)	SNO_3_ (mg kg^−1^)	pH
Low		13426 ± 1796.38 a	25.50 ± 8.39 a	5.28 ± 1.46 a	20.32 ± 8.17 a	13.98 ± 0.84 a	957.33 ± 154.93 a	18.18 ± 0.45 a	13.93 ± 0.05 a	7.18 ± 0.03 a	4.61 ± 0.02 a	6.46 ± 0.20 a
High		12647 ± 2243.24 b	22.79 ± 9.04 b	3.54 ± 2.72 b	15.95 ± 8.61 b	13.40 ± 1.83 b	870.28 ± 181.25 b	17.16 ± 0.96 b	13.40 ± 0.04 b	6.54 ± 0.02 b	3.03 ± 0.02 b	6.47 ± 0.02 a
	N0	10463.93 ± 304.40 d	12.84 ± 0.45 d	1.93 ± 0.16 d	6.78 ± 0.16 d	12.27 ± 0.64 c	713.97 ± 42.58 d	14.83 ± 0.10 d	11.72 ± 0.03 e	6.29 ± 0.02 d	1.61 ± 0.02 e	6.73 ± 0.04 a
	N200	11836.25 ± 156.63 c	19.61 ± 0.31 c	3.16 ± 0.14 c	13.08 ± 0.39 c	12.92 ± 0.24 bc	810.38 ± 27.55 c	17.39 ± 0.05 c	13.16 ± 0.03 d	6.56 ± 0.02 c	2.33 ± 0.02 d	6.56 ± 0.02 b
	N250	12950.22 ± 365.29 b	23.20 ± 0.51 b	4.41 ± 0.44 b	20.44 ± 2.07 b	13.21 ± 0.67 b	879.99 ± 65.16 b	17.85 ± 0.12 b	13.93 ± 0.06 c	6.70 ± 0.02 b	3.60 ± 0.02 c	6.41 ± 0.05 c
	N300	14951.01 ± 243.78 a	33.11 ± 0.31 a	6.18 ± 0.28 a	25.34 ± 0.48 a	15.34 ± 0.46 a	1092.5 ± 59.54 a	19.13 ± 0.03 a	14.61 ± 0.04 b	7.41 ± 0.02 a	5.51 ± 0.02 b	6.31 ± 0.02 d
	N350	14981.67 ± 202.22 a	31.97 ± 0.80 a	6.37 ± 0.39 a	25.03 ± 0.68 a	14.70 ± 0.49 a	1072.21 ± 68.38 a	19.18 ± 0.09 a	14.92 ± 0.05 a	7.31 ± 0.03 a	6.07 ± 0.01 a	6.30 ± 0.04 d

SHN_4_, soil ammonium; SNO_3_, soil nitrate; SOC, soil organic carbon; STN, soil total nitrogen; S_UR, urease activities; S_ACP, acidic phosphates activities; S_AI, acid invertase activities; S_β-GC, Beta-glucosidase activities; S_CAT, catalase activities; and S_CL, cellulase activities. Different letters within the same column denote significant differences (p < 0.05) among the soil samples.

**Table 2:** Pearson’s correlation analysis for soil enzymes and physio-chemical properties.

	S_ACP	S_AI	S_ β_GC	S_CAT	S_CL	SNH_4_	SNO_3_	SOC	STN	S_UR
S_AI	0.9806									
S_ β_GC	0.9096	0.8853								
S_CAT	0.9607	0.9548	0.9316							
S_CL	0.8803	0.8802	0.8498	0.8276						
SNH_4_	0.8496	0.8393	0.7781	0.804	0.7573					
SNO_3_	0.9218	0.9077	0.8389	0.8644	0.8270	0.9202				
SOC	0.9316	0.9227	0.9026	0.9419	0.7993	0.7258	0.8426			
STN	0.8989	0.9168	0.8433	0.9131	0.7628	0.7744	0.8408	0.9553		
S_UR	0.9476	0.9471	0.8841	0.9113	0.8655	0.8279	0.9111	0.8791	0.8803	
pH	−0.8959	−0.9233	−0.7618	−0.8876	−0.7548	−0.6863	−0.8049	−0.9174	−0.8975	−0.8458

SHN_4_, soil ammonium; SNO_3_, soil nitrate; SOC, soil organic carbon; STN, soil total nitrogen; S_UR, urease activities; S_ACP, acidic phosphates activities; S_AI, acid invertase activities; S_*β*_GC, beta-glucosidase activities; S_CAT, catalase activities; and S_CL, cellulase activities.

### Changes in Bacterial OTUs Numbers, Richness, and Diversity

A total of 2,186 bacterial OTUs were identified from 1891700 RNA gene sequencing. A total of 1,292,546 reads of high quality bacterial RNA gene were obtained after screening, with an average of 43,084 reads per sample. The bacterial OTU number exhibited the trend N300 > N250 > N200 > N350 > N0 under low irrigation regimes, and N350 > N300 > N250 > N200 > N0 under high irrigation regimes (Supplementary Figures S1A,B). The OTUs in each sample were filtered at a minimum count of 2 and 10% prevalence. After filtering, a total of 2,394 and 2,490 OTUs were obtained in which all the treatments shared 2,100 and 2,063 OTUs under low and high irrigation regimes, respectively (Supplementary Figures S1A,B). The bacterial community richness and diversity showed significant differences among the different N fertilizer dosages and irrigation regimes (3). The diversity showed the trend N300 > N350 > N250 > N200 > N0 and the richness showed the trend N300 > N250 > N350 > N200 > N0. The N300 treatment under low irrigation markedly increased the richness and diversity of the soil bacterial communities.

### Bacterial Alpha and Beta Diversity

**Figure 1:**
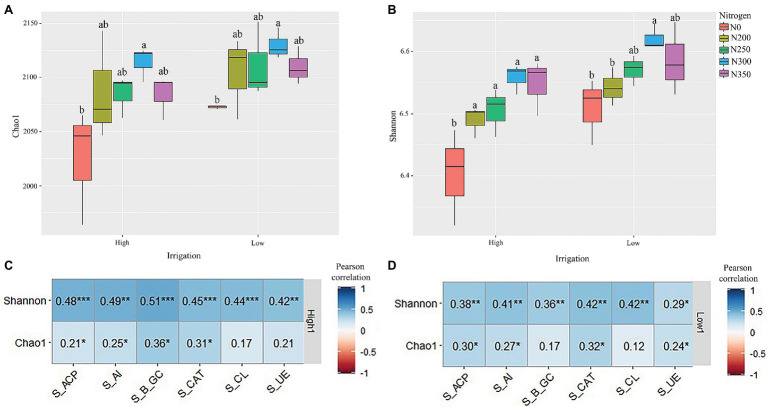
**(A,B)** Bacterial alpha diversity indices represented by richness (a), and Shannon diversity (b) in each soil compartment and variety. **(C,D)** The correlation between indices of bacterial alpha diversity in high irrigation **(C)** and low irrigation **(D)** and environmental factors using Pearson correlation analysis. ^*^p < 0.05; ^**^p < 0.01; and ^***^p < 0.01.

The indexes of bacterial OTU Chao1 richness and Shannon diversity were used to signify bacterial alpha diversity. These indices across the nitrogen dosages were lower under high irrigation water treatment than lower irrigation water treatment (Figures 1A,B). Additionally, the indices of bacterial OTU Chao1 richness and Shannon diversity were different among different fertilizer dosages in both high and low irrigation regimes. These results suggest that the N300 had significantly higher Chao1 and Shannon indexes compared to the other N treatments (*p* < 0.001). Pearson correlation analysis indicated that Shannon indexes of both high and low irrigation for S_ACP, S_AI, S_β_GC, S_CAT, S_CL, and S_UE were significantly positively correlated (*p* < 0.05), likewise, S_ACP, S_AI, S_β_GC, and S_CAT were positively correlated for Chao1 in high irrigation and S_ACP, S_AI, S_CAT, and S_UE in low irrigation regimes (Figures 1C,D).

**Figure 2:**
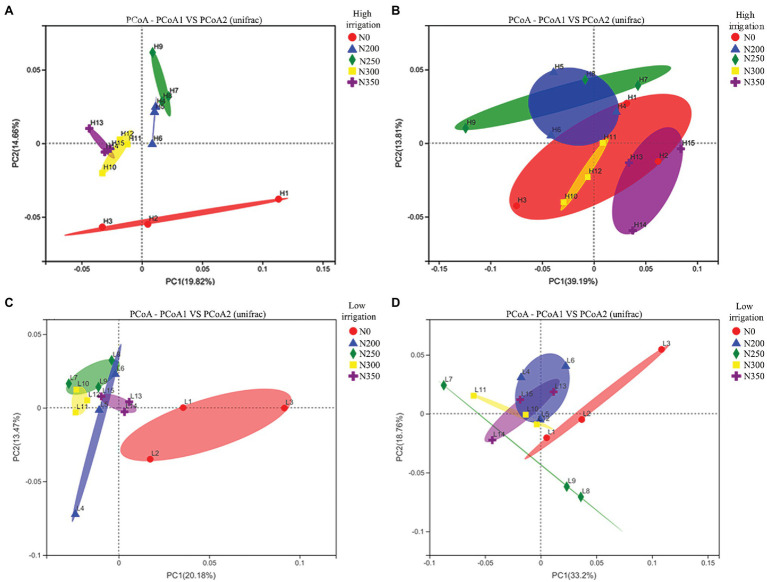
Principal coordinate analyses (PCoAs) using unweighted UniFrac metric **(A,C)** and weighted UniFrac metric **(B,D)** indicate that the largest separation between bacterial communities is spatial distribution of irrigation water (PCo1) and the second largest source of variation is nitrogen fertilizer (PCo2).

To assess bacterial beta diversity, phylogenetic analysis of bacterial compositions were performed using unweighted (Figures 2A,C) and weighted UniFrac distances (Figures 2B,D). The findings revealed that low irrigation had a more comparable community structure than high irrigation. The correlations among N200, N250, N300, and N350 under low irrigation treatments were closer than those of high irrigation, despite being significantly different from N0 (Figures 2A,C). Likewise, closer correlations were noted between N200 and N250 as well as N300 and N350 but were away from N0 treatment (Figure 2A). After weighting, there were significant differences among N250, N300, and N350 under high irrigation treatment (Figure 2B), but no significant differences between N200, N300, and N350 under low irrigation regimes were noted (Figure 2D).

### Bacterial Community Composition and Abundance

The dominant bacterial phyla with a relatively greater abundance (>1%) and high contributions are listed in Table 1. Proteobacteria (23.02%) had the highest relative abundance among the bacterial community, followed by Actinobacteriota (20.84%), Chloroflexi (14.88%), Firmicutes (14.02%), Acidobacteriota (9.18%), Gemmatimonadota (4.69%), Myxococcota (3.46%), Methylomirabilota (2.59%), and Bacteroidota (1.11%; Supplementary Figure S2; Supplementary Table S1). The two major classes of Proteobacteria were Alpha-proteobacteria (68%) and Gamma-proteobacteria (32%; Supplementary Figure S3). Acidimicrobiia (17.20%), Thermoleophilia (24.70%), Actinobacteria (52.90%), and MB_A2_108 (4.80%) were highly abundant classes of Actinobacteriota (Supplementary Figure S3). Similarly, the highly abundant classes of phyla Chloroflexi were Chloroflexia (32.62%), Anaerolineae (21.23%), Dehalococcoidia (12.36%), and JG30_KF-CM66 (10.91%) whereas, Bacilli is a highly prominent class of the phyla Firmicutes and is dominating by 92.96% relative abundance of the phyla Firmicutes, followed by Clostridia (6.41%). Different N fertilizer rates and irrigation had a significant effect on the dominant bacterial species. Our results demonstrated that the Proteobacteria were the most abundant bacterial phyla in the soil microbiome, dominating in N250 and N0 under high and low irrigation regimes, respectively (Supplementary Figure S2). Whereas the Actinobacteriota had a higher relative abundance in the N0 and N200 under high and low irrigation regimes, respectively (Supplementary Figure S2).

**Figure 3:**
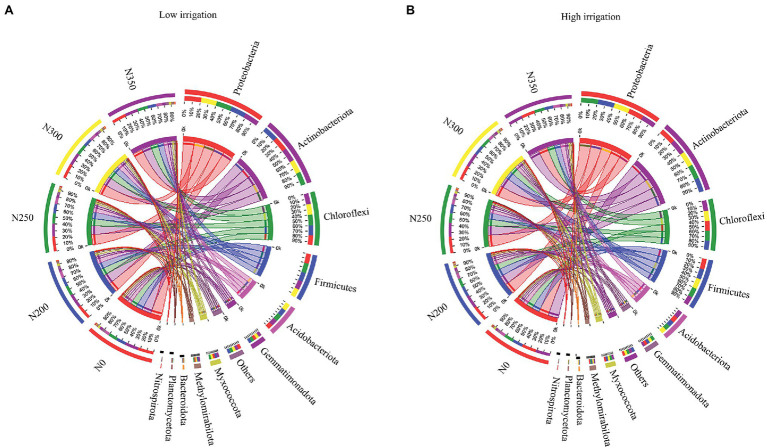
Bacterial composition and abundance in the soil under low irrigation **(A)**, and high irrigation **(B)**. Dominant bacterial phyla are shown in Circos maps.

In the N0, N200, N250, N300, and N350 soils, Proteobacteria, Actinobacteria, Chloroflexi, and Firmicutes were the top four most abundant phyla irrespective of irrigation regimes (Figures 3A,B). In the N250 and N300 soils, the Proteobacteria (23%), Actinobacteria (19%), and Chloroflexi (16%) were the top three most abundant phyla in the low irrigation regime (Figure 3A). The abundances of Proteobacteria and Acidobacteriota were noticeably increased, whereas the abundances of Actinobacteria and Gemmatimonadota were markedly decreased in the N300 and N250 soils compared with the N350 soil. In addition, the Proteobacteria and Firmicutes were potentially increased, whereas the abundances of Chloroflexi and Acidobacteriota were decreased in the N0 compared to the N350 soil (Figure 3A). The above results suggest that soils with optimum N fertilization (N250 and N300 soils) present a significantly greater abundance of Chloroflexi and Acidobacteriota in low irrigation regimes. Similarly, in high irrigation regimes, the Proteobacteria (23%), Actinobacteria (22%), Chloroflexi (15%), and Firmicutes (15%) were the top four most abundant phyla in N300 soil (Figure 3B). Our results further suggest that the Proteobacteria (25%) were markedly increased in the N250 soil, whereas the Actinobacteria (21%), Chloroflexi (14%), and Firmicutes (14%) were decreased compared to N300. Soil with high irrigation regimes in N250 had significantly higher abundances of Proteobacteria and Actinobacteria but lower abundances of Chloroflexi.

At the genus level, the bacterial community structures showed significant differences among the different N dosages and irrigation regimes (Supplementary Figures S4A,B). For example, in the N250 and N300 soils, the most dominant genera were *Bacillus* (22%) and *Vicinamibacteraceae* (23%), respectively (Supplementary Figure S4A). *Pedomicrobium*, *MND1*, *Streptomyces*, and *Nocardioides* were the most dominant genera and significantly increased in the N0 soil compared to other N treatments. In high irrigation regimes, the N0, N200, and N300 highest abundance of genera of *Bacillus* (24%), *Pedomicrobium* (24%), and *MND1* (22%), respectively (Supplementary Figure S4B). In addition, the Streptomyces was significantly higher in the N300 soil compared to the other N treatments. These results indicated that the abundances of the genera *Bacillus* increased with increase of N dose (N250) under the low irrigation regimes, however, decreased under high irrigation regimes (Supplementary Figures S4A,B). The rarefaction curve demonstrates that all of the samples had greater sequencing depth and diversity (Supplementary Figure S5).

**Figure 4:**
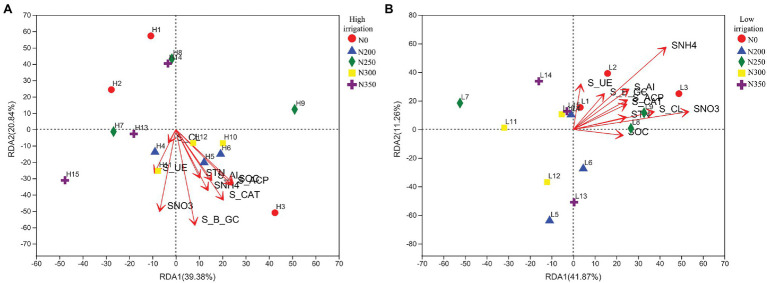
Redundancy analysis of different zones, abundant classes, and ten environmental factors (arrows) indicates the dominant communities and influential environmental factors in the soil under low irrigation **(A)**, and high irrigation **(B)**.

Redundancy analysis was used to understand the relationship between environmental conditions and bacterial composition. These results demonstrated that the first two axes of RDA accounted for 20.84 and 39.38%, respectively, of the total variation of the data in high irrigation regime (Figure 4A), while 11.26 and 41.87% in low irrigation regimes (Figure 4B). Soil mineral N (SNO_3_^−^-N and SNH_4_^+^-N) and enzymes were much more closely associated with the bacterial community distribution in N200 and N300 treatments. In addition, S_UE and SNO_3_^−^-N were correlated to the bacterial community distribution in N350 s under high irrigation. While in low irrigation, SOC, STN, SNO_3_^−^-N, and SHN_4_^+^-N along with soil enzymes were correlated with N0 and N200.

### Relationship of the Bacterial Community With Environmental Factors

**Figure 5:**
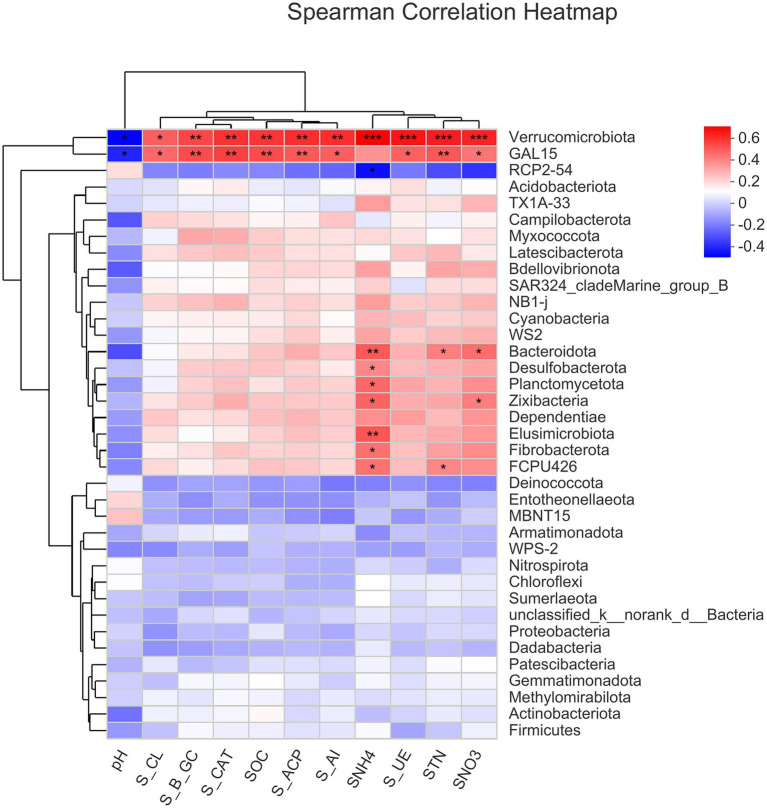
Spearman correlation analyses show the bacterial phyla that are significantly positively/negatively correlated with soil enzymes and physicochemical parameters in the soils. ^*^0.01 *p* ≤ 0.05, ^**^0.001 *p* ≤ 0.01, and ^***^p ≤ 0.001.

**Figure 6:**
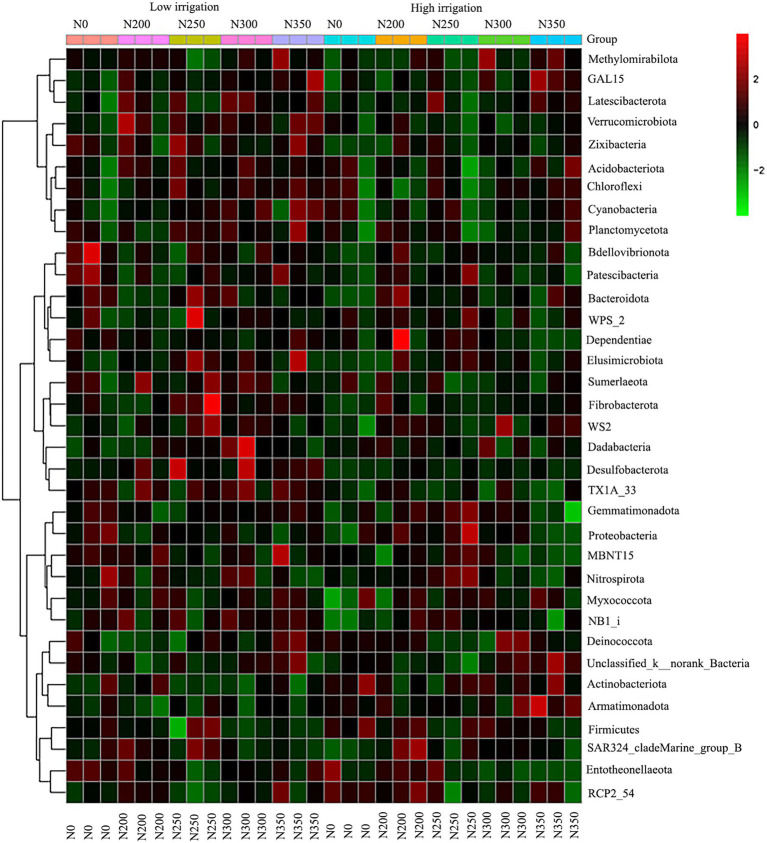
Microbial community composition of top 50 abundant operational taxonomic units (OTUs) and their abundance level with cluster analysis. The dendrogram linkages and OTUs distances are not phylogenetic but based upon the reads number. The legend and scale represent the abundance of OTUs (*Y*-axis) within each sample (*X*-axis). The red indicates a more abundant phyla and green represents less abundant phyla in the corresponding samples.

The correlation heat map of different bacterial phyla showed a positive correlation of Verrucomicrobiota and GAL15 with soil enzyme activities (S_ACP, S_AI, S_β_GC, S_CAT, S_CL, and S_UE) and soil chemical properties (SNH_4_^+^-N, SNO_3_^−^-N, SOC, and STN), but negatively correlated with soil pH. Moreover, there was a significantly positive correlation of SNH_4_ with Zixibacteria, Planctomycetota, Desulfobacterota, Bacteroidota, Elusimicrobiota, Fibrobacterota, and FCPU426 while it was significantly negatively correlated with RCP2-54 (Figure 5). Based on the constructed correlation heat map, most of the bacterial phyla were correlated with soil enzymes and soil physio-chemical properties. Moreover, STN was significantly positively correlated with Bacteroidota and FCPU426 while Bacteroidota and Zixibacteria phyla were significantly positively correlated with SNO_3_^−^-N (Figure 5). The data on community composition for each bacterial phyla were clustered based on abundance distribution or degree of similarity. The bacterial phyla, N dosages, and irrigation regimes were graded independently and presented using heat maps based on the clustering results. Color gradients were adopted to differentiate between phyla with high and low abundance and to highlight the N dosage similarities in community composition (Figure 6).

## Discussion

Soil nutrients, microorganisms, and enzyme, are important components of the agricultural eco-system and believed to be affected by irrigation and nitrogen management (Chen et al., 2019; He and Wang, 2021). Irrigation management is considered to have a strong impact on the soil microbial communities, structures, and composition (He and Wang, 2021). The soil enzyme activities, STN, SOC, SNO_3_^−^-N, and SNH_4_^+^-N were significantly higher in low irrigation compared to high irrigation regimes (Table 1). Similarly, the soil enzyme activities were also significantly increased with increasing N fertilization till 300 kg N ha^−1^. It is well documented that moderate water can reduce the loss of available nutrients in the soil, promote the absorption and utilization of nutrients, and thus increase soil fertility and microorganisms (Peralta et al., 2014; Zeng et al., 2016). He and Wang (2021) demonstrated that the sustainable use and management of irrigation water and nutrients are crucial. Our results are in line with the previous findings of Morugán-Coronado et al. (2019), who demonstrated that nutrient estimates in microbial biomass suggested greater N and P immobilization in moderate water deficit plots (Zhou et al., 2021). The correlation analysis showed that the soil enzyme activities were significantly correlated with SNH_4_^+^-N, SNO_3_^−^-N, SOC, and STN (Table 2). Our results agree with the previous literature reported by Morugán-Coronado et al. (2019), demonstrated that higher soil water content can promote microbial growth and metabolic enzyme activities, which may increase N utilization efficiency and lower STN.

Soil water content is the main driving force shaping soil microbial communities, structure, and activity (Guenet et al., 2012). Microbial diversity is important for ecosystem constancy and soil productivity because it regulates soil chemical processes (Chen et al., 2015) and is required for agricultural sustainability (Ferris and Tuomisto, 2015). The effects of N fertilizer rates and irrigation regimes on bacterial alpha diversity and richness were significant (Figures 1A,B). In both irrigation regimes, the N300 treatment had significantly higher richness compared to other N treatments, and was attested by Chao1 (*p* < 0.05; Figure 1A). The direct addition of nutrients to soils, altering soil pH, and stimulating soil decomposition as a result of N fertilization has strongly affected the bacterial communities (Beare et al., 2002). In addition, the bacterial diversity was estimated by the Shannon index (*p* < 0.05), suggesting that the N300 treatment had significantly higher bacterial diversity under a low irrigation regime than the other treatments in the group (Figure 1B). The increase in bacterial richness and diversity under low irrigation might be due to more N being available to soil microbes as compared to the lower N availability under high irrigation, which might have N losses due to leaching (Khajanchi et al., 2015). There is evidence that optimum wetting improves the bacterial diversity, communities, and structure in soil, while excessive drying and wetting have the opposite effect (Peralta et al., 2014). These results are in line with the previous literature reported by Colombo et al. (2016) and Li et al. (2021), who reported that the amount of irrigation water is an important management factor in assessing the soil microbial community diversity.

The correlation analysis showed that soil enzymes were positively correlated with bacterial diversity under high irrigation (Shannon index; Figure 1C), but S_CL and S_UE were not significantly correlated with bacterial richness (Chao1 index; Figure 1C). However, the soil enzymes had direct and positive correlation with bacterial richness under low irrigation regimes (Figure 1D), except S_β_GC and S_CL. Nitrogen fertilizer is known to increase microbial diversity over time, these results are in conformity with the findings of earlier studies (Ma et al., 2020). Zeng et al. (2016) reported that nitrogen fertilizer and irrigation regimes can have a considerable impact on soil microbial richness, diversity, and the activity of enzymes in soil (Ming et al., 2018). The alpha and beta diversity were significantly and favorably associated with N fertilization and irrigation (Figures 1A,B) based on unweighted UniFrac distances (Figures 2A,C). Compared to high irrigation, the bacterial diversity was much more closely related to N fertilizer under low irrigation. After weighting the species abundance there were significant differences among N250, N300, and N350 under high irrigation (Figure 2B) as well as with N200, N300, and N350 under low irrigation regimes (Figure 2D). The greater N availability for to soil microbes under low irrigation with moderate N fertilization may better explain this scenario (Mack et al., 2005). However, the losses of N with high irrigation water as a result of leaching may need more fertilization. Furthermore, these changes in bacterial diversity could be explained by the quality and amount of irrigation water and root exudates (Sawicka et al., 2020; Wang et al., 2021c), which are the most important sources of nutrients for rhizosphere bacteria. Primary metabolites including amino acids, carbohydrates, and organic acids (Hou et al., 2020), secreted by the plants roots have a positive effect on C and N concentrations, as well microbial population (Frenk et al., 2015, 2018).

The results of the current study indicated that irrigation and N fertilization had much stronger effects on the bacterial community and composition (Supplementary Figure S2; Table 1). It was observed that the Proteobacteria were the dominant phyla in all the treatments of both irrigation regimes (Figure 3). Our results are in line with the previous published studies (Bei et al., 2018; Li et al., 2021). Different irrigation water and N fertilizer treatments changed soil physio-chemical qualities, which are habitually linked to soil bacterial communities. Li et al. (2021) also reported that the soil physiognomies and chemical properties are considered an important variable affecting the bacterial abundance. The effects of N fertilization and irrigation on bacterial richness and diversity were found significant, however, the interaction between irrigation and N were not significant (Supplementary Figure S2 Table 3). These results are in conformity with findings of by Shen et al. (2010), who found that N dosages and irrigation water had a strong effect on soil bacterial community structure. In our results, the bacterial community structure at genus level showed a significant difference with N fertilization and irrigation (Supplementary Figure S4). Phylum and OTU-level RDA analyses showed that soil mineral N (SNO_3_^−^-N and SNH_4_^+^-N), STN, and SOC were correlated with bacterial communities in both irrigation regimes, suggesting that there was a strong correlation between the soil enzyme activities and the nutrient contents in the soil which affecting the bacterial communities (Figure 4). The bacterial phyla Proteobacteria are Gram-negative bacteria that are extremely vulnerable to environmental disruption and irrigation water (Schimel et al., 2007). Our results are in line with the findings of Dennis et al. (2010), who reported that the relative abundance of Proteobacteria significantly increases with an increase in irrigation water. However excessive wetting and drying has a negative effect on the soil bacterial community (Peralta et al., 2014) as a result of certain rhizomatous sediments production under high irrigation treatment.

**Table 3:** Effect of different treatments and the interaction between irrigation water and N fertilization rate on the richness and diversity of the bacteria community.

Water	Nitrogen	Shannon	Simpson	Ace	Chao1	Coverage (%)
Low		6.57 ± 0.05 a	0.0039 ± 0.001 a	2093.90 ± 30.86 a	2105.40 ± 28.75 a	99.58 ± 0.08 a
High		6.50 ± 0.07 b	0.0034 ± 0.001 b	2063.60 ± 44.06 b	2078.80 ± 42.90 b	99.50 ± 0.09 b
	N0	6.46 ± 0.08 d	0.0042 ± 0.001 a	2032.10 ± 48.49 b	2048.59 ± 42.88 b	99.45 ± 0.10 b
	N200	6.52 ± 0.04 c	0.0038 ± 0.001 ab	2082.65 ± 36.16 a	2095.33 ± 50.43 a	99.57 ± 0.04 a
	N250	6.54 ± 0.05 bc	0.0035 ± 0.001 b	2085.40 ± 35.66 a	2097.88 ± 29.16 a	99.52 ± 0.11 ab
	N300	6.59 ± 0.04 a	0.0034 ± 0.001 b	2109.25 ± 20.28 a	2121.83 ± 15.85 a	99.60 ± 0.04 a
	N350	6.57 ± 0.05 ab	0.0034 ± 0.001 b	2084.44 ± 23.08 a	2096.74 ± 20.27 a	99.56 ± 0.11 a
Interactions						
	Water	***	**	*	*	**
	Nitrogen	***	**	**	**	*
	Water * Nitrogen	ns	ns	ns	ns	ns

At *p* > 0.05, ns (not significant).

* 0.01 < *p* ≤ 0.05;

** 0.001 < *p* ≤ 0.01; and

*** *p* ≤ 0.001.

Different letters within the same column denote significant differences (*p* < 0.05) among the soil samples.

Many previous studies have shown that irrigation water management and fertilizer N greatly influence microbial communities in the soil (Mack et al., 2005; Gheysari et al., 2009), enzymatic activities (Ming et al., 2018). The present study showed that the bacterial phyla Verrucomicrobiota and GLA15 were positively correlated with soil enzymes and physio-chemical properties (*p* > 0.001; Figure 5). However, the bacterial phyla RCP2-54 had negatively correlated with NH_4_^+^-N. The results of the present study suggest that the N fertilizer and irrigation management had positive effects on soil nutrient contents and soil enzymes, which showed positive and direct correlation with bacterial phyla Chloroflexi and Cyanobacteria (Beare et al., 2002; Ming et al., 2018). The N fertilization under both irrigation regimes showed that the species richness and sequence sample size increased with increasing N dosages (Supplementary Figure S5). Sufficient soil water is a benchmark and indicates an adequate nutrient availability due to dissolving many types of nutrients. However, too high N dosages, on the other hand, have irrevocable consequences, permanently affecting bacteria and even causing death (Li et al., 2021).

## Conclusion

This study investigates the impact of irrigation water and N fertilizer on the composition and diversity of bacterial communities, as well as their effects on soil enzymes and the physio-chemical properties of soil under maize plantation. Nitrogen fertilization enhanced the soil bacterial community, diversity, richness, and number of sequences epically under low irrigation regimes. Low irrigation regimes boosted soil enzymes (S_ACP, S_AL, S_β_GC, S_Cl, and S_UR), STN, SOC, SNH_4_^+^-N, and SNO_3_^−^-N. The N300 treatment had significantly higher bacterial richness, diversity, and soil enzyme activities. The relative abundance of Protobacteria, Actinodacteriota, Chlorofexi, and Firmicutes in response to irrigation and N management influenced the bacterial composition, richness, and communities. These findings will be helpful in framing the irrigation and N fertilization management systems for improving the soil fertility, bacterial structure, and communities on sustainable way and minimizing the negative impacts of environmental pollution in sub-tropical regions of the China.

## Data Availability Statement

The datasets presented in this study can be found in online repositories. The names of the repository/repositories and accession number(s) can be found at: NCBI – PRJNA792847.

## Author Contributions

IM: conceptualization and writing-original draft preparation. IM and LY: methodology and formal analysis. SA, SF, and MZ: investigation. XZ: resources and supervision. IM and AK: data curation. AK: writing-review and editing. All authors contributed to the article and approved the submitted version.

## Funding

This study was financially supported by the National Natural Science Foundation of China (31760354) and the Natural Science Foundation of Guangxi Province (2019GXNSFAA185028).

## Conflict of Interest

The authors declare that the research was conducted in the absence of any commercial or financial relationships that could be construed as a potential conflict of interest.

## Publisher’s Note

All claims expressed in this article are solely those of the authors and do not necessarily represent those of their affiliated organizations, or those of the publisher, the editors and the reviewers. Any product that may be evaluated in this article, or claim that may be made by its manufacturer, is not guaranteed or endorsed by the publisher.
