# Supplementary material for: Irrigation and Nitrogen Fertilization Alter Soil Bacterial Communities, Soil Enzyme Activities, and Nutrient Availability in Maize Crop
Source: Front Microbiol. 2022 Feb 3;13:833758. doi: 10.3389/fmicb.2022.833758 (PMC8851207; doi:10.3389/fmicb.2022.833758)
Supplement: Supplementary file 1 [file Data_Sheet_1.docx]

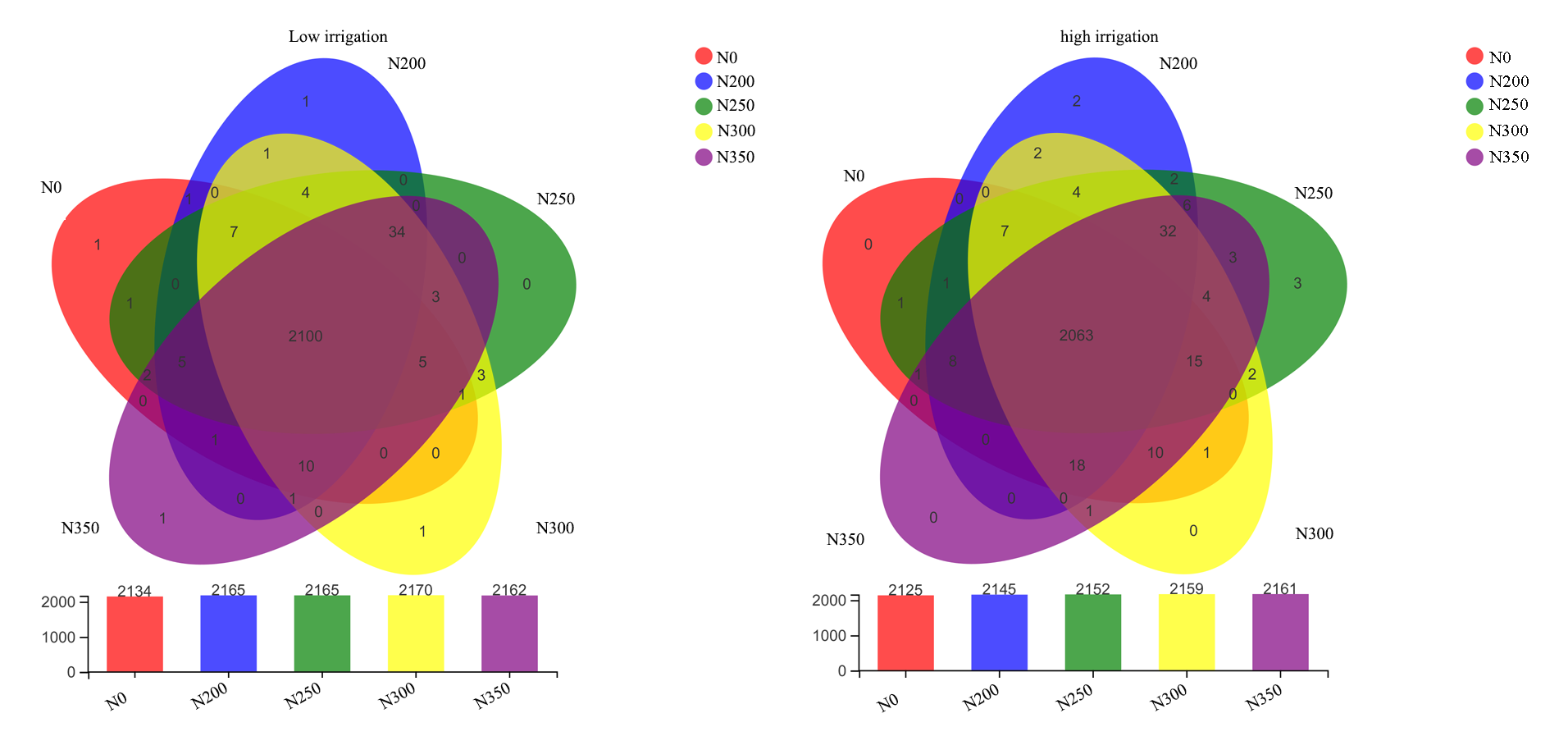


Figure S1. Venn diagram of bacterial phyla in the five N rates under low irrigation (a), and high irrigation (b).


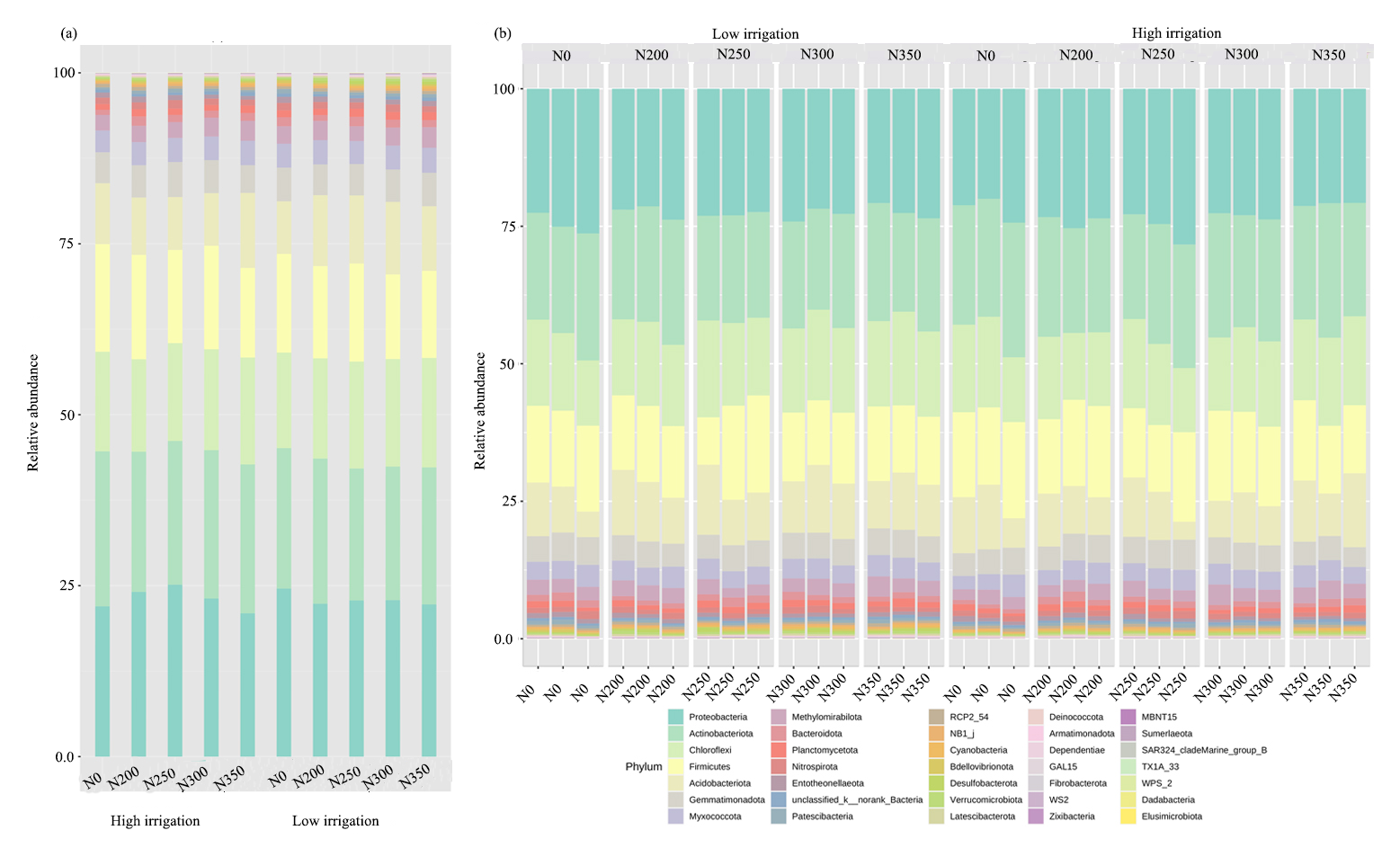


Figure S2. Relative abundance of different phyla in each treatment (average of three replicate) under low and high irrigation (a) and relative abundance in each replicate (b).


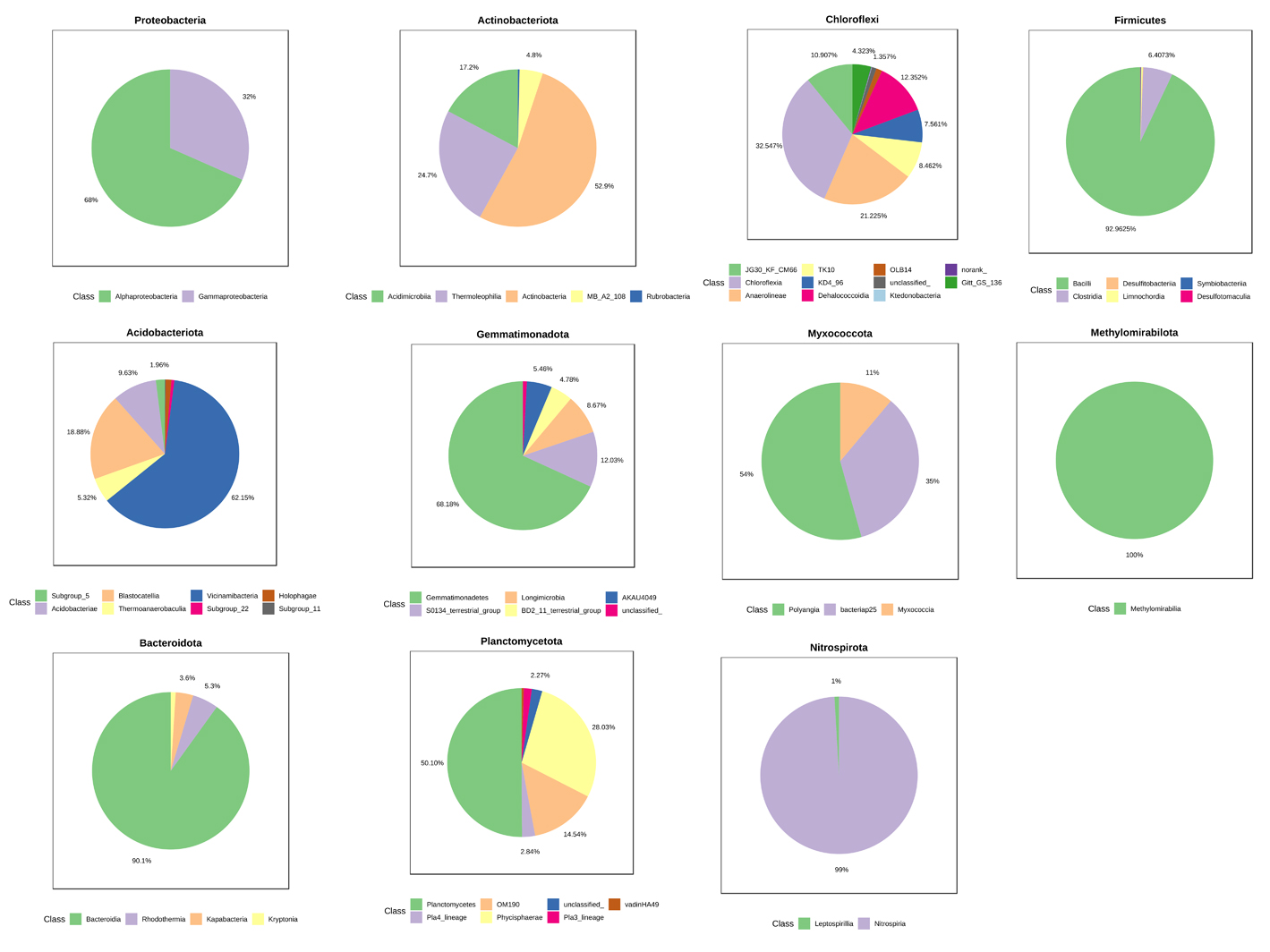


Figure S3. The relative abundance of major classes from the top 11 abundant bacterial phyla.


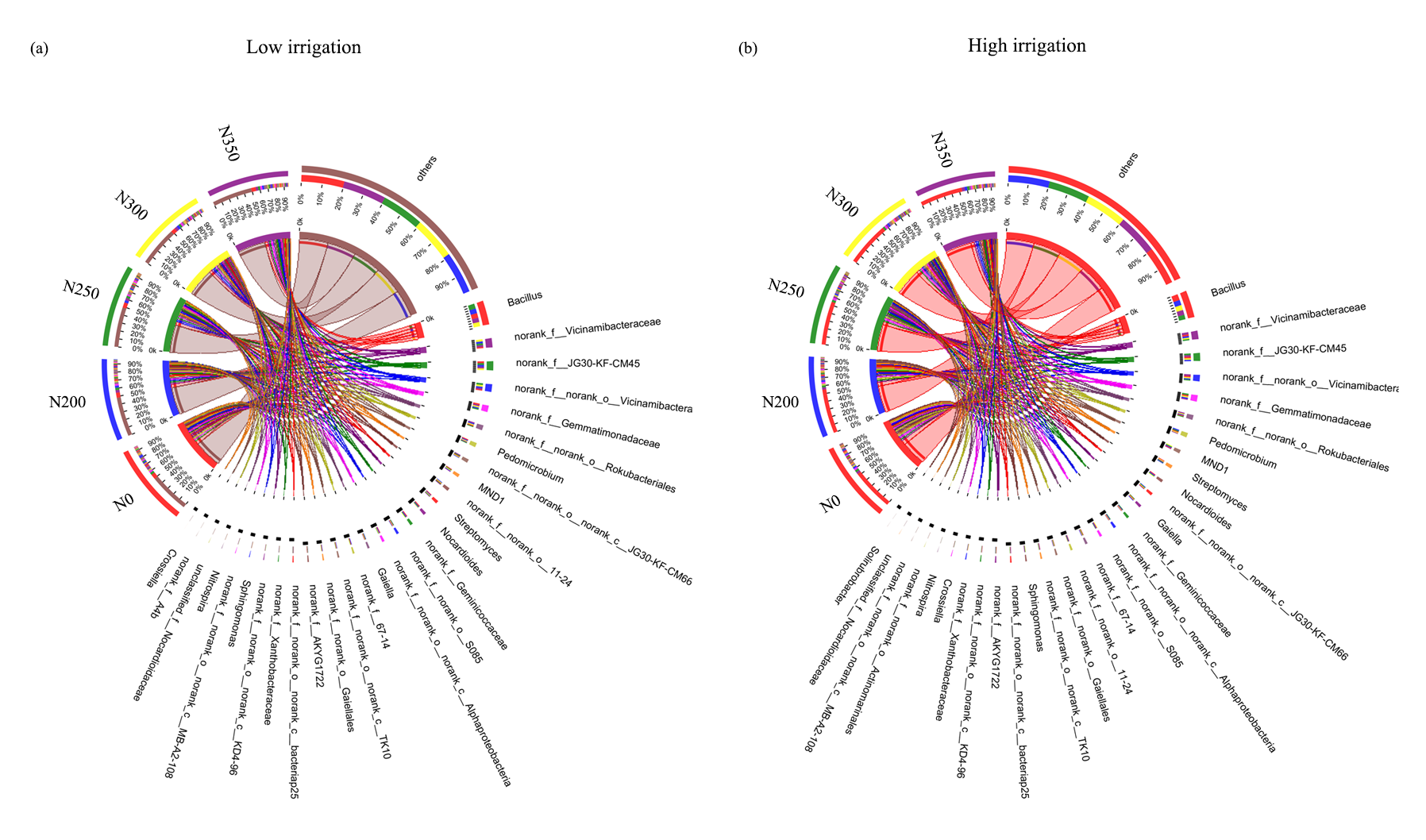


Figure S4. Bacterial composition and abundance in the five N rates under low irrigation (a), and high irrigation (b). Dominant bacterial genera are shown in Circos maps.


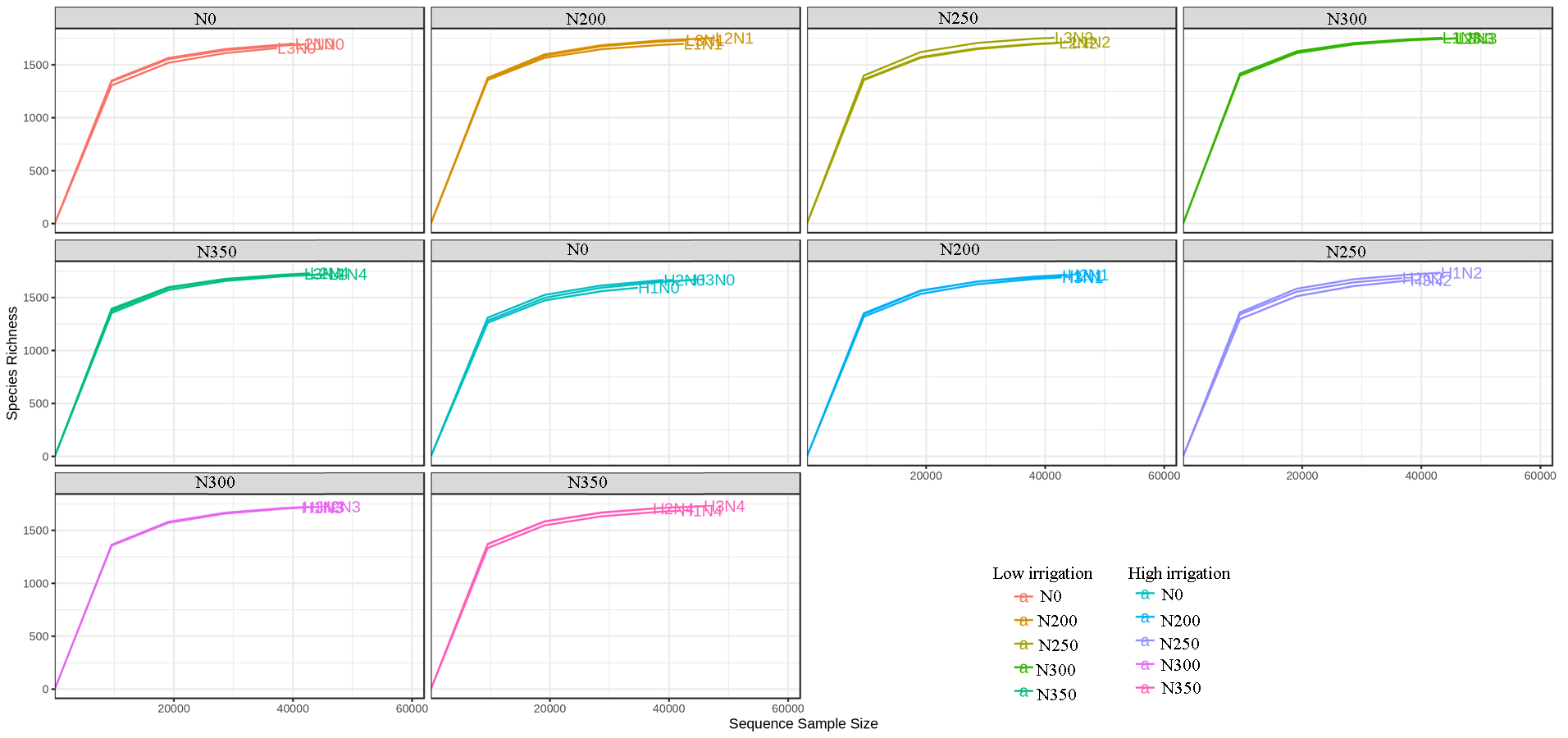


Figure S5. Rarefaction curve depicting species richness and number of sequences. Each sub figure represents the different N treatment under different irrigation.

Table S1. Effect of irrigation and N fertilization rates on top 11 soil bacterial phyla abundance in maize crop.

| Treatments | | Planctomycetota | | Proteobacteria | Actinobacteriota | Chloroflexi | Firmicutes | Acidobacteriota | | Gemmatimonadota | | Myxococcota | Methylomirabilota | Bacteroidota | Nitrospirota | Others |
| --- | --- | --- | --- | --- | --- | --- | --- | --- | --- | --- | --- | --- | --- | --- | --- | --- |
| High | N0 | 0.83 | 21.97 | | 22.67 | 14.57 | 15.74 | | 8.91 | | 4.53 | 3.17 | 2.28 | 0.76 | 0.93 | 3.63 |
|  | N200 | 1.06 | 24.09 | | 20.51 | 13.52 | 15.26 | | 8.39 | | 4.72 | 3.36 | 2.40 | 1.38 | 0.99 | 4.32 |
|  | N250 | 0.93 | 25.14 | | 21.02 | 14.31 | 13.63 | | 7.74 | | 5.13 | 3.51 | 2.32 | 1.05 | 1.24 | 3.98 |
|  | N300 | 0.85 | 23.12 | | 21.70 | 14.76 | 15.14 | | 7.69 | | 4.83 | 3.46 | 2.75 | 1.02 | 0.92 | 3.77 |
|  | N350 | 1.085 | 20.95 | | 21.79 | 15.63 | 13.095 | | 10.98 | | 4.05 | 3.56 | 2.92 | 1.15 | 0.86 | 3.93 |
| Low | N0 | 1.01 | 24.57 | | 20.56 | 13.97 | 14.42 | | 7.69 | | 4.93 | 3.49 | 2.56 | 1.33 | 1.09 | 4.39 |
|  | N200 | 0.91 | 22.35 | | 21.24 | 14.66 | 13.48 | | 10.37 | | 4.51 | 3.55 | 2.81 | 0.85 | 1.00 | 4.26 |
|  | N250 | 1.19 | 22.84 | | 19.30 | 15.64 | 14.34 | | 9.97 | | 4.59 | 3.34 | 2.17 | 1.39 | 0.91 | 4.33 |
|  | N300 | 1.13 | 22.88 | | 19.54 | 15.73 | 12.37 | | 10.61 | | 4.74 | 3.49 | 2.64 | 1.15 | 1.09 | 4.63 |
|  | N350 | 1.15 | 22.258 | | 20.045 | 16.01 | 12.73 | | 9.46 | | 4.89 | 3.65 | 3.01 | 1.04 | 0.87 | 4.89 |
| **Average** | | **1.01** | **23.02** | | **20.84** | **14.88** | **14.02** | | **9.18** | | **4.69** | **3.46** | **2.59** | **1.11** | **0.99** | **4.21** |
